# Immune Modulation of Metastatic Niche Formation in the Bone

**DOI:** 10.3389/fimmu.2021.765994

**Published:** 2021-10-20

**Authors:** Xinyu Cheng, Zhan Wang

**Affiliations:** ^1^ Department of Neonatology, The Children’s Hospital, Zhejiang University School of Medicine, National Clinical Research Center for Child Health, Hangzhou, China; ^2^ Department of Orthopedic Surgery, The Second Affiliated Hospital, Zhejiang University School of Medicine, Hangzhou, China; ^3^ Orthopedics Research Institute of Zhejiang University, Hangzhou, China; ^4^ Key Laboratory of Motor System Disease Research and Precision Therapy of Zhejiang Province, Hangzhou, China

**Keywords:** bone metastasis, disseminated tumor cells, immune cells, chemokines, cytokines

## Abstract

Bone metastasis is commonly seen in patients with breast cancer, prostate cancer and lung cancer. Tumor-intrinsic factors and the tumor microenvironment cooperate to affect the formation of bone metastatic niche. Within the bone microenvironment, immune cells have been regarded as a major contributor to metastatic progression. In this review, we describe the dynamic roles of immune cells in regulating metastatic homing, seeding, dormancy, and outgrowth in the bone. We also summarize the diverse functions of immune molecules including chemokines, cytokines, and exosomes in remodeling the bone metastatic niche. Furthermore, we discuss the therapeutic and prognostic potential of these cellular and molecular players in bone metastasis.

## Introduction

**Figure 1:**
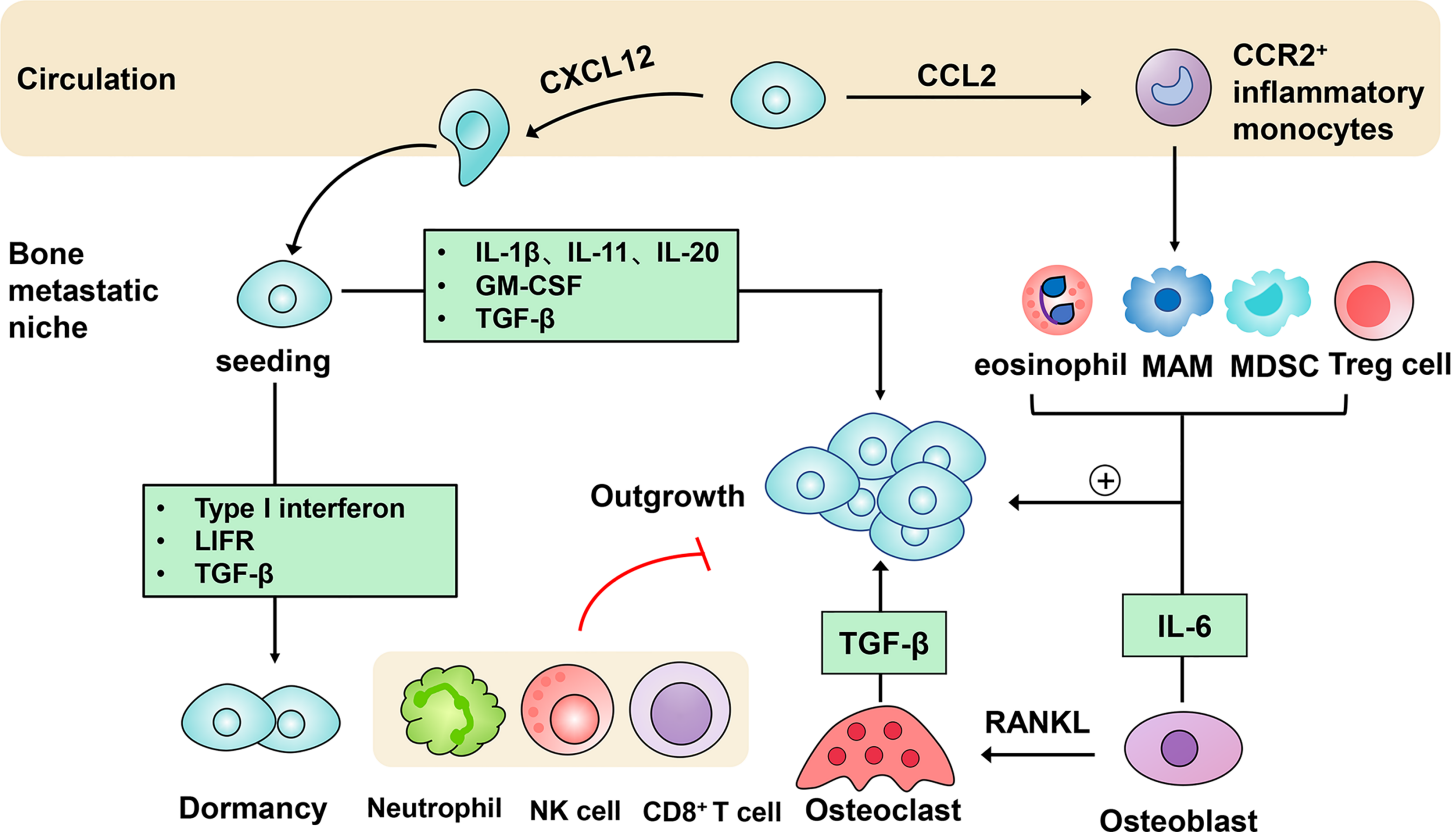
Immune mechanisms in the progression of bone metastasis. After being detached from the primary tumor, circulating tumor cells produce CXC-chemokine ligand 12 (CXCL12) to facilitate metastatic homing to the bone. They can also secrete CC-chemokine ligand 2 (CCL2) to recruit CC-chemokine receptor 2 (CCR2)^+^ inflammatory monocytes towards the bone metastatic niche, in which they differentiate into metastasis-associated macrophages (MAMs). Following metastatic seeding into the bone, disseminated tumor cells can remain in a dormant state driven by type I interferon, the receptor of leukemia inhibitory factor (LIFR), transforming growth factor-β (TGF-β). Induced by several immune molecules, dormant tumor cells can be reactivated and initiate metastatic outgrowth. Eosinophils, MAMs, myeloid-derived suppressor cells (MDSCs), regulatory T cells (Tregs) conspire to support the persistent growth of metastatic tumor cells. Furthermore, the tumor-stroma interactions are engaged in the acceleration of bone metastasis. Osteoblasts-derived receptor activator of nuclear factor-κB-ligand (RANKL) induces the differentiation of osteoclasts, which in turn stimulates bone resorption and tumor cell proliferation mediated by growth factors like transforming growth factor-β (TGF-β). In addition, immune cells including neutrophils, nature killer (NK) cells and CD8^+^ T cells act to restrict metastatic tumor cell growth.

Bone metastasis is a multi-stage process which involves escape from the primary site, survival in the circulation and metastatic colonization (1). These events are driven by both cancer cell-intrinsic traits and extrinsic factors in the tumor microenvironment (TME) (2). Emerging evidence has highlighted the importance of immune modulation in the bone metastatic niche formation (3–6). In this review, we aim to focus on the contribution of immune cellular components and molecular mediators to the homing, seeding, dormancy and subsequent outgrowth of metastatic cancer cells in the bone microenvironment (**Figure 1**).

## Immune Cells Modulating Metastatic Niche Formation in the Bone

### Innate Immune Cells

Macrophages participate in all steps of metastasis. They can be polarized into either proinflammatory M1-like macrophages or anti-inflammatory M2-like macrophages in response to environmental stimuli (7). In the primary site, tumor-associated macrophages (TAMs) are involved in angiogenesis, migration and intravasation (8). At the distant organs, metastasis-associated macrophages (MAMs), a distinct population of TAMs, were first described in mouse models of breast cancer lung metastasis (9). They were reported to promote tumor cell extravasation, seeding and metastatic outgrowth (10, 11). Similar to lung MAMs, bone MAMs also play metastasis-promoting roles. Our recent study reveals that MAMs are enriched in mouse models of breast cancer bone metastasis and patient samples. Macrophage depletion with clodronate liposomes and colony-stimulating factor 1 receptor (CSF1R) reduces breast cancer cell growth in the bone. Using lineage tracing methods and gene expression analysis, we are the first to demonstrate bone MAMs originate from circulating inflammatory monocytes and bear specific cell surface markers CD204 and interleukin 4 receptor (IL4R). IL4R acts as promoters of metastatic outgrowth. The absence of IL4R in bone MAMs results in a reduction of metastatic tumor growth in the bone, which holds therapeutic promise for bone metastatic patients (12).

Neutrophils, a key player in the innate immunity, have gained attention in the metastatic process (13–15). The neutrophil-to-lymphocyte ratio in the blood has been reported as a prognostic indicator of survival in patients with bone metastasis (16). Moreover, neutrophil accumulation in the TME showed resistance to immune checkpoint blockade (ICB) relative to other immune compositions (17). Given the highly heterogeneity and plasticity of neutrophils, they display opposing behaviors during the metastatic dissemination and colonization. On one hand, neutrophils elicit cytotoxic responses by directly killing tumor cell to suppress metastatic growth. In the bone metastatic prostate cancer, Ly6G^+^ neutrophils are recruited to the bone lesions and induce apoptosis of prostate cancer cells through inhibition of signal transducer and activator of transcription (STAT) 5. Notably, the cytotoxic property of neutrophils can be gradually lost during tumor progression, which is consistent with previously reported immunosuppressive activity of neutrophils in the late stages of cancer (18, 19). On the other hand, increasing evidence indicates the relevance of neutrophils in supporting metastatic establishment in other distal organs. For example, neutrophils are shown to facilitate extravasation of tumor cells and lung colonization through secretion of inflammatory cytokines and suppression of natural killer (NK) cells (20). Overall, these studies uncover the context-dependent roles of neutrophils in metastasis.

NK cells are innate cytotoxic lymphocytes that mediate cancer cell killing in the tumor progression (21). In the experimental metastasis models of breast cancer, metastatic cancer cells have been found to evade the NK cell immune attack and remain dormancy by downregulating levels of ligands for NK-cell activating receptors (22). The functions of NK cell can also be impaired by certain signaling pathways and inflammatory cytokines in bone metastasis. A recent study indicated that inhibition of the JAK/STAT pathway dramatically decreased NK cell activity and enhanced the metastatic burden, which could be restored by interleukin-15 (IL-15) stimulation (23).

While initially regarded as an anti-tumor immune component (24), eosinophils in the context of inflammation also contribute to bone metastasis. Mechanistically, they secrete CC-chemokine ligand 6 (CCL6) to attract tumor cells to the bone metastatic niche through CC-chemokine receptor 1 (CCR1). Inhibition of the CCL6-CCR1 signaling significantly attenuates the tumor cell migration and bone metastasis formation, which suggests the close connection between inflammation and metastasis and provides new paradigms for cancer prevention (25). Furthermore, plasmacytoid dentritic cells have been described in mouse models of breast cancer bone metastasis. Their metastasis-supporting effects are driven by induction of immunosuppressive cells and osteolytic cytokine receptor activator of nuclear factor-κB-ligand (RANKL) (26).

### Adaptive Immune Cells

CD8^+^ T cells exert cytotoxic effects in the bone metastatic niche. They act as negative regulators of tumor growth in the bone. Adoptive transfer of cytotoxic CD8^+^ T cells into PLCγ2^-/-^ mice prevents the growth of B16 melanoma cells in the bone (27). Their anti-tumor responses in the bone are further enhanced by the transcription factor estrogen-related receptor alpha (ERRα). On one hand, overexpression of ERRα increased the production of the chemokines CCL17 and CCL20 to attract more cytotoxic T cells the bone. On the other hand, ERRα decreased the tumor cell secretion of anti-inflammatory cytokine transforming growth factor-β (TGF-β) (28). In contrast to CD8^+^ T cells, cancer-primed CD4^+^ T cells are reported to create the premetastatic niche *via* the release of RANKL before bone colonization in breast cancer models (29). As a subtype of CD4^+^ T cells, the immunosuppressive regulatory T cells (Tregs) enrichment has been detected in the bone marrow of patients with prostate cancer. These cells are found to inhibit the differentiation of osteoclasts (OCs), which might elucidate the potential mechanisms of osteoblastic lesions of prostate cancer (30).

### Other Non-Immune Cells

Myeloid-derived suppressor cells (MDSCs) represent a heterogenous population of immature myeloid cells with immunosuppressive features (31). An abundance of MDSCs is observed in both preclinical mouse models and patients with bone metastasis (32, 33). Several factors are required for the accumulation and function of MDSCs in the bone microenvironment. Interferon regulatory factor 7 (IRF7) was reported to suppress pro-metastatic activity of MDSCs in the 4T1 murine metastatic model. Overexpression of IRF7 reduced metastatic burden and prolonged survival time by counteracting the action of MDSCs and restoring CD8^+^ T cell and NK cell activity (34). More recently, DKK1, an inhibitor of the Wnt pathway, has been directly targets β-catenin in MDSCs from mouse models and patients. Neutralization of DKK1 leads to reduced tumor growth and MDSC expansion (35). Additionally, since MDSCs are progenitors of macrophages, they also have the capacity to differentiate into bone-resorbing OCs in a nitric oxide-dependent manner, thereby enhancing bone destruction and tumor growth (36).

## Immune Mediators Remodeling the Bone Metastatic Niche

### Chemokines

Increasing evidence reveals the involvement of chemokines in the bone metastatic progression. In breast cancer, CCL2 induces the recruitment of circulating inflammatory monocytes to the bone through the interaction with CCR2 (12). Similarly, prostate cancer-derived CCL2 also facilitates tumor growth in the bone, which results from increased OC differentiation and angiogenesis (37). Treatment with anti-CCL2 antibody attenuated metastatic outgrowth and prolonged survival in mouse models of breast cancer metastasis (12). However, application of anti-CCL2 agents alone should be cautioned because discontinuation of CCL2 inhibition has rebound effects that augment metastasis in mice (38). Furthermore, inhibition of CCR2 with humanized monoclonal antibodies, MLN1202, has shown beneficial effects in patients with bone metastases, as evidenced by reduced levels of bone-resorbing factors urinary N-telopeptide of type I collagen (uNTX) (ClinicalTrials. gov ID: NCT01015560). CCL4 binding to CCR5 on bone marrow fibroblasts stimulates their release of connective tissue growth factor/CCN2, which supports breast cancer cell growth in bone (39). High expressions of CCL5 are associated with high Gleason grade and poor prognosis in prostate cancer patients (40). In response to prostate cancer cell growth-induced pressure, osteocytes produce CCL5, which promotes tumor proliferation in the bone (41). CXC-chemokine ligand 5 (CXCL5) are also reported to deliver growth signals to malignant cells. Exposure to TME elicits CXCL5 secretion from bone marrow cells, which allows breast cancer cells to exit from dormancy and colonize the bone microenvironment (42). The CXCL12/CXC-chemokine receptor 4 (CXCR4) axis plays an important role in regulating DTC homing and survival. In triple negative breast cancer (TNBC), CXCL12 from cancer-associated fibroblasts is found to select cancer cells with high Src activity homing to the bone (43). Moreover, the CXCL12/CXCR4 pathway modulates tumor growth in the bone. In prostate cancer, pyruvate kinase M2 (PKM2) released by primary cancer-derived exosomes initiates the downstream release of hypoxia inducible factor-1α (HIF-1α) and CXCL12, which promotes metastatic seeding and outgrowth. Genetic inhibition and pharmacological blockade of CXCR4 diminish the number of bone metastases and total metastatic burden (44). This study suggests that the growth of tumor cells in the distant organs can be conditioned by metabolic alterations at the primary site.

### Cytokines

Interferons (IFN), comprised of type I IFNs and type II IFNs (IFNγ), are critical cytokines during the anti-metastatic immune response (45). The importance of type I IFNs in the bone metastatic niche was shown in three non-bone metastatic breast cancer models in which loss of host type I IFN signaling accelerated metastatic spreading to the bone in part through impairing the ability of NK cells to eliminate cancer cells (46). As observed in breast cancer, tumor-inherent type I IFN was also downregulated in proliferative prostate cancer cells from bone, which disrupted dormancy status and promoted metastatic outgrowth. This effect could be reversed by the use of histone deacetylase inhibitors (HDACi), suggesting the therapeutic potential of epigenetic modifications in the regulation of IFN signaling (47). Importantly, the presence of IRF9 is a positive prognostic biomarker for the chemotherapeutic response in TNBC patients (48). In addition, IFN-γ can directly target OCs to inhibit bone loss and bone metastasis (49).

Interleukin-1β(IL-1β) is highly expressed in tumor cells and bone marrow cells during progression of bone metastasis. IL-1β in tumor cells induces epithelial to mesenchymal transition (EMT) and correlates with recurrence and bone relapse in patients with stage II and III breast cancer. Bone marrow-derived IL-1β can activate intracellular nuclear factor Kappa B (NF-κB) and the Wnt signaling to promote metastatic expansion to form overt metastases. Targeting IL-1β with anti-IL-1β antibody or the IL-1 receptor antagonist, Anakinra, prevents metastatic seeding and colony formation (50, 51). IL-6 is a critical promoter of tumor growth in osteolytic bone metastasis of breast cancer. In this process, tumor cell-derived Jagged1 simulates Notch signaling in osteoblasts, which increases the IL-6 release and OC differentiation (52). IL-11, a member of the IL-6 family, has a similar effect in osteolytic lesions dependent on the STAT3 signaling (53). By contrast, the receptor of leukemia inhibitory factor (LIFR), whose ligand LIF belongs to the IL-6 family of cytokines, shows an anti-tumor effect through induction of dormancy in the bone (54). IL-20 is a tumor-promoting cytokine partly due to their capacity to induce proliferation and migration of tumor cells. Mice treated with anti-IL-20 antibody 7E displayed a reduction in bone colonization and osteolysis (55). Mesenchymal stem cell-derived IL-28 confers apoptotic resistance properties on prostate cancer cells disseminated to bone lesions *via* STAT3 activation (56). Although initially identified as an inhibitor of osteoclastogenesis (57), granulocyte macrophage-colony stimulating factor (GM-CSF), produced by breast cancer cells, has been recently found to increase the number of OCs and bone metastatic potential (58). Another cytokine TGF-β is involved in bone metastasis. TGF-β released by the bone matrix is a well-established driver of tumor growth and bone degradation (59). However, osteoblast-derived TGF-β induces dormancy of metastatic prostate cancer cells in the bone mediated by TGFβR-III-p38MAPK-pS249/T252RB pathway (60). This demonstrates the temporal differences of TGF-β signaling during different stages of bone metastasis. Beyond stromal cells, cancer cells can also express TGF-β to suppress the Wnt signaling through dishevelled binding antagonist of β-catenin 1(DACT1)- dependent biochemical condensates formation, which in turn promotes bone metastatic outgrowth (61). Finally, in the mouse model of prostate cancer bone metastasis, high levels of TGF-β in the TME were associated with poor response to ICB. This immunosuppressive effect of TGF-β is achieved by restraining type 1 T helper cell development. The efficacy of ICB was enhanced by TGF-β inhibition, suggesting the potential value of targeting TGF-β to improve ICB response (62).

### Exosomes

Exosomes are nano-sized extracellular vesicles secreted by multiple cell types. They deliver signaling molecules such as proteins, lipids, microRNAs (miRNAs) to specific target cells, affecting the bone metastatic formation (63, 64). Breast cancer-derived exosomes containing CCL3, CCL27 and other molecules are found to remodel the bone microenvironment, characterized by stimulation of osteoclastogenesis and angiogenesis (65). Similarly, exosomes from melanoma cells can reprogram the phenotype of bone marrow progenitor cells and support tumor metastasis to the bone (66). In addition, exosomes from stromal cells contribute to the dormancy of breast cancer cells *via* reducing CXCL12 levels (67). Stromal-derived exosomal miR-148a-3p was reported to target the ERK1/2 signaling to suppress the proliferation of bone metastatic cancer cells (68). Furthermore, plasma exosomes act as noninvasive biomarkers to predict response to Radium-223 treatment. In bone metastatic prostate cancer, exosomal programmed death-ligand 1 (PD-L1) was upregulated in patients with unfavorable response to radium-223 treatment (69).

## Perspectives

Immune cells exhibit either pro-metastatic or anti-metastatic activity in the bone microenvironment. Their reciprocal interactions with tumor cells and other bone-resident cells are essential for the bone metastatic progression. Technological advances have allowed us to identify specific immune cell subtypes and define their molecular characteristics. Blocking the pro-tumoral phenotypes of immune cells and exploiting their anti-tumor activities represent a novel therapeutic strategy to prevent the formation and recurrence of bone metastasis. In addition, immune mediators including chemokines, cytokines, and exosomes have shown great therapeutic and prognostic value in the bone metastases. Furthermore, the bone microenvironment can also influence the metastatic seeding into other distant organs triggered by epigenetic reprogramming (70). Despite deep insights into the immune landscape of bone metastasis, future studies are still needed to identify novel determinants and elucidate their immunological mechanisms, which will pave the way for immune-based therapy and improve clinical outcomes.

## Author Contributions 

XC wrote the manuscript. ZW revised the manuscript. All authors contributed to the article and approved the submitted version.

## Funding

This work was supported by the China Postdoctoral Science Foundation (2021M692792) and National Natural Science Foundation of China (82103499).

## Conflict of Interest

The authors declare that the research was conducted in the absence of any commercial or financial relationships that could be construed as a potential conflict of interest.

## Publisher’s Note

All claims expressed in this article are solely those of the authors and do not necessarily represent those of their affiliated organizations, or those of the publisher, the editors and the reviewers. Any product that may be evaluated in this article, or claim that may be made by its manufacturer, is not guaranteed or endorsed by the publisher.
